# Building a Digital Health Research Platform to Enable Recruitment, Enrollment, Data Collection, and Follow-Up for a Highly Diverse Longitudinal US Cohort of 1 Million People in the All of Us Research Program: Design and Implementation Study

**DOI:** 10.2196/60189

**Published:** 2025-01-15

**Authors:** Dave Klein, Aisha Montgomery, Mark Begale, Scott Sutherland, Sherilyn Sawyer, Jacob L McCauley, Letheshia Husbands, Deepti Joshi, Alan Ashbeck, Marcy Palmer, Praduman Jain

**Affiliations:** 1 Vibrent Health, Inc Fairfax, VA United States; 2 Boston VA Research Institute VA Boston Health Care Veterans Administration Boston, MA United States; 3 John P. Hussman Institute for Human Genomics Miller School of Medicine University of Miami Miami, FL United States; 4 The Task Force for Global Health, Inc Decatur, GA United States

**Keywords:** longitudinal studies, cohort studies, health disparities, minority populations, vulnerable populations, precision medicine, biomedical research, decentralization, digital health technology, database management system

## Abstract

**Background:**

Longitudinal cohort studies have traditionally relied on clinic-based recruitment models, which limit cohort diversity and the generalizability of research outcomes. Digital research platforms can be used to increase participant access, improve study engagement, streamline data collection, and increase data quality; however, the efficacy and sustainability of digitally enabled studies rely heavily on the design, implementation, and management of the digital platform being used.

**Objective:**

We sought to design and build a secure, privacy-preserving, validated, participant-centric digital health research platform (DHRP) to recruit and enroll participants, collect multimodal data, and engage participants from diverse backgrounds in the National Institutes of Health’s (NIH) All of Us Research Program (AOU). AOU is an ongoing national, multiyear study aimed to build a research cohort of 1 million participants that reflects the diversity of the United States, including minority, health-disparate, and other populations underrepresented in biomedical research (UBR).

**Methods:**

We collaborated with community members, health care provider organizations (HPOs), and NIH leadership to design, build, and validate a secure, feature-rich digital platform to facilitate multisite, hybrid, and remote study participation and multimodal data collection in AOU. Participants were recruited by in-person, print, and online digital campaigns. Participants securely accessed the DHRP via web and mobile apps, either independently or with research staff support. The participant-facing tool facilitated electronic informed consent (eConsent), multisource data collection (eg, surveys, genomic results, wearables, and electronic health records [EHRs]), and ongoing participant engagement. We also built tools for research staff to conduct remote participant support, study workflow management, participant tracking, data analytics, data harmonization, and data management.

**Results:**

We built a secure, participant-centric DHRP with engaging functionality used to recruit, engage, and collect data from 705,719 diverse participants throughout the United States. As of April 2024, 87% (n=613,976) of the participants enrolled via the platform were from UBR groups, including racial and ethnic minorities (n=282,429, 46%), rural dwelling individuals (n=49,118, 8%), those over the age of 65 years (n=190,333, 31%), and individuals with low socioeconomic status (n=122,795, 20%).

**Conclusions:**

We built a participant-centric digital platform with tools to enable engagement with individuals from different racial, ethnic, and socioeconomic backgrounds and other UBR groups. This DHRP demonstrated successful use among diverse participants. These findings could be used as best practices for the effective use of digital platforms to build and sustain cohorts of various study designs and increase engagement with diverse populations in health research.

## Introduction

### Background

Longitudinal cohort studies have historically relied on clinic-based recruitment models that limit participation and reduce cohort diversity in many ways [1,2]. System-level barriers, such as distance to the research site and clinic-based eligibility, can negatively affect participant recruitment and study retention. Socioeconomic barriers, such as income, education, and health insurance status, also limit research participation in minority and other health-disparate groups [3-5]. The resultant homogeny in research cohorts reduces the generalizability and validity of research outcomes [6-8]. Digital platforms can minimize barriers to research participation, such as transportation costs, site access, and time commitment, and significantly improve participant access and engagement [9-11]. As a result, digital platforms are being increasingly used in health research to enable accelerated, more accessible, and more reliable real-world data collection. However, as longitudinal cohorts increase in the breadth and scale of data, the efficacy of digital platforms can vary profoundly, depending on the platform’s capability to meet the ever-increasing needs of the study over time.

Appropriate implementation of study activities via digital platforms can minimize many of the commonly reported barriers to research participation. Recent studies, such as MyHeart Counts and Health eHeart, show how digital enablement of traditional research methods may improve engagement of people from diverse backgrounds, including those who do not commonly engage with health care systems [12-14]. Digital technologies also reduce the stakeholder burden related to collection, curation, and sharing of health data [15]. Therefore, digital platforms may be effective tools to improve participant engagement, increase cohort diversity, broaden geographical reach, and streamline the curation of diverse research data sets.

Digital research platforms must also accommodate users of varying digital aptitudes. This requires consideration of sociodemographic factors that impact the use of digital technology, such as age, disability, rurality, education, income, culture, digital access, native language, and literacy [16,17]. Digital research platforms can be adapted to accommodate these differences and thereby reduce participant burden and build trust with communities that are underrepresented in health research [18]. Existing commercial and academic tools for electronic data capture (EDC) are built primarily for research teams and are not designed to meet the preferences of participants interacting with them [19]. Therefore, it is important to create digital tools with engaging functionality for diverse participants that also meet the evolving needs of longitudinal cohort studies. We aimed to build such a platform to support nationwide enrollment of 1 million diverse participants in a novel multiyear, longitudinal cohort study.

### Objectives

**Figure 1:**
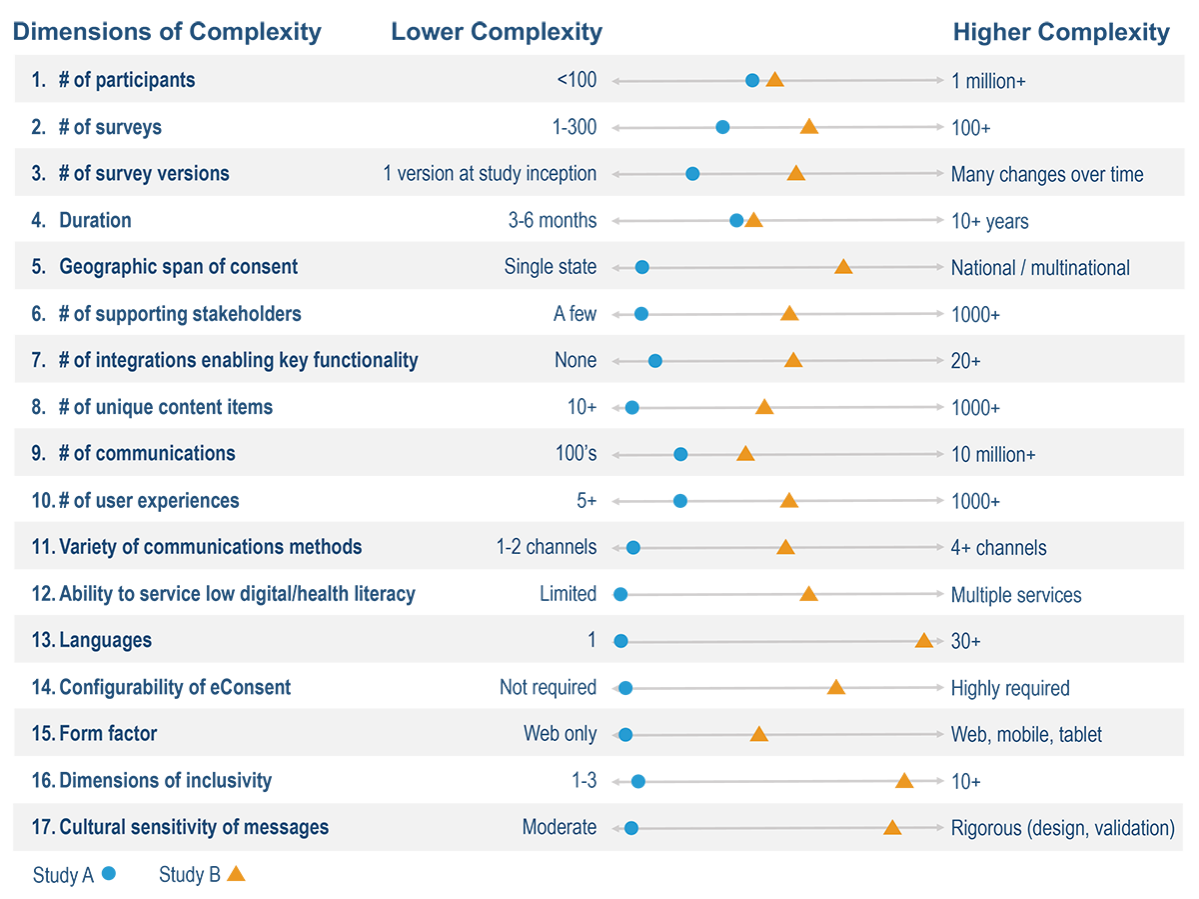
Illustration of how an all-in-one platform requires broad functionality to accommodate the varying dimensions of complexities for different study protocols.

In this paper, we describe various tools within a digital health research platform (DHRP) designed to effectively engage diverse participants from various backgrounds and collect multisource health data in a large community-based longitudinal cohort study. The All of Us Research Program (AOU) is an ongoing nationwide initiative aimed to recruit 1 million participants from diverse cultural, socioeconomic, demographic, and geographic backgrounds and to collect data that are generalizable, accelerate biomedical research, and improve outcomes for all groups [20,21]. The broadly inclusive eligibility, recruitment, and data curation needs of the AOU required the development of a highly adaptable, comprehensive digital platform able to accommodate multifaceted, complex study requirements. The study also needed a platform with modifiability that would allow study staff across 1200 sites to conduct operations and rapidly deliver customized, community-specific engagement (Figure 1). These complex needs led to the development of a comprehensive digital platform design capable of supporting data expansion and the addition of ancillary studies over the extended study timeline of 10 or more years [22].

## Methods

### System Requirements

**Figure 2:**
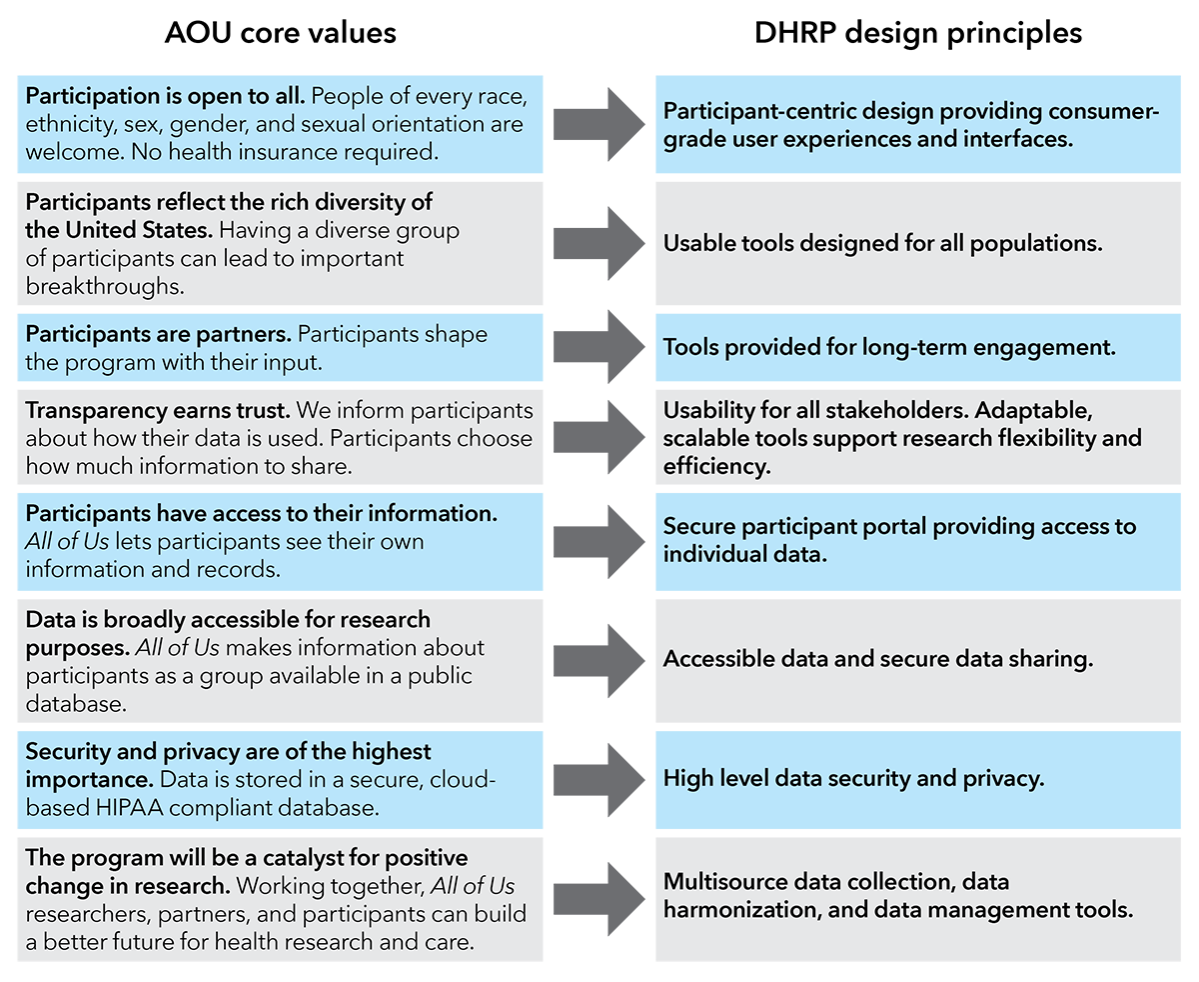
AOU core values were used to develop key design features of the DHRP. AOU: All of Us Research Program; DHRP: digital health research platform; HIPAA: Health Insurance Portability and Accountability Act.

Vibrent Health, Inc, a digital health technology company, was selected as the participant technology systems center and tasked with developing a secure digital platform and tools for participant experience, study management, and data analysis [23]. To align with its core values, the AOU needed a secure DHRP that (1) could be broadly deployed nationwide to support study management, (2) could host several program touch points within a flexible participant journey, (3) was accessible to different levels of digital access, literacy, and comfort, and (4) was cybersecure and robust (Figure 2).

### System Description

#### Digital Health Research Platform Design and Architecture

**Figure 3:**
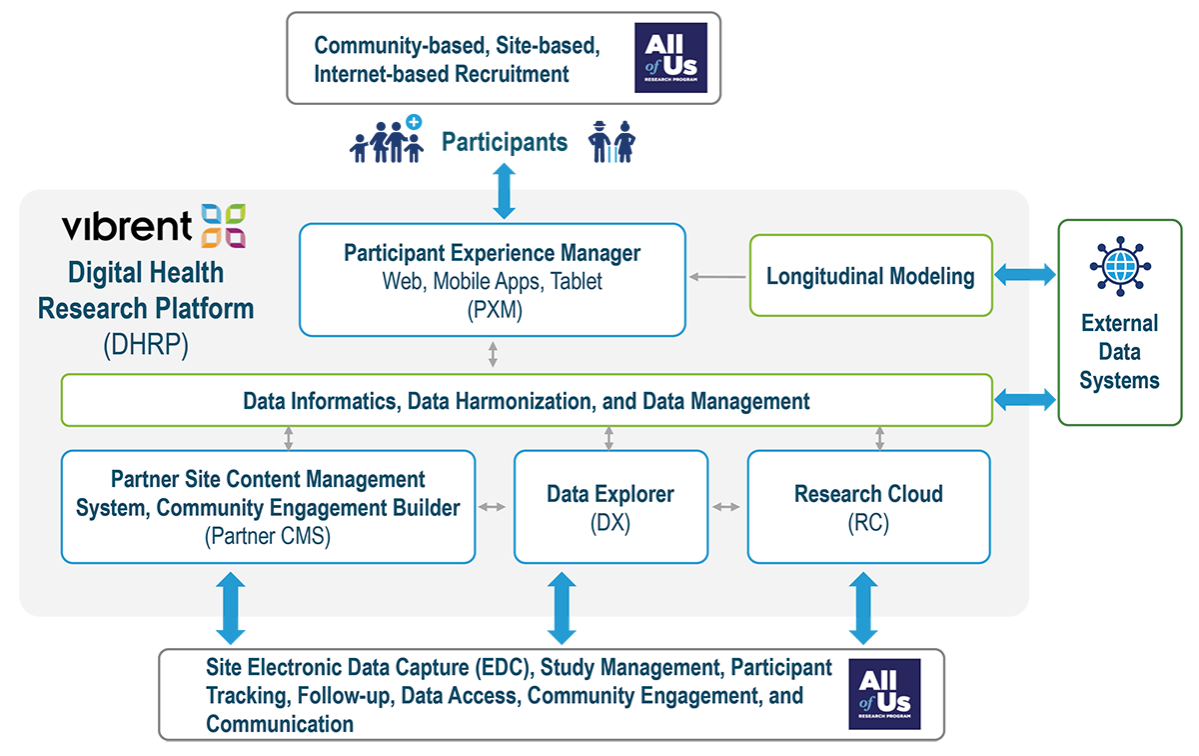
Integration of DHRP tools within the AOU digital infrastructure. AOU: All of Us Research Program; DHRP: digital health research platform; EDC: electronic data capture.

Vibrent Health designed and built a DHRP to facilitate participant recruitment, enrollment, multisource data collection, and long-term engagement using web-capable digital devices (computers, mobile devices, tablets). The DHRP was developed via close collaboration between the government, academia, community stakeholders, and industry using validated user experience (UX) and user interface (UI) research. (Figure 3).

We developed participant-facing tools for engagement and data collection, as well as researcher-facing study management tools. The data collected within the DHRP were stored in a cybersecure cloud environment, where they could be harmonized, cleaned, and integrated with other data systems.

**Figure 4:**
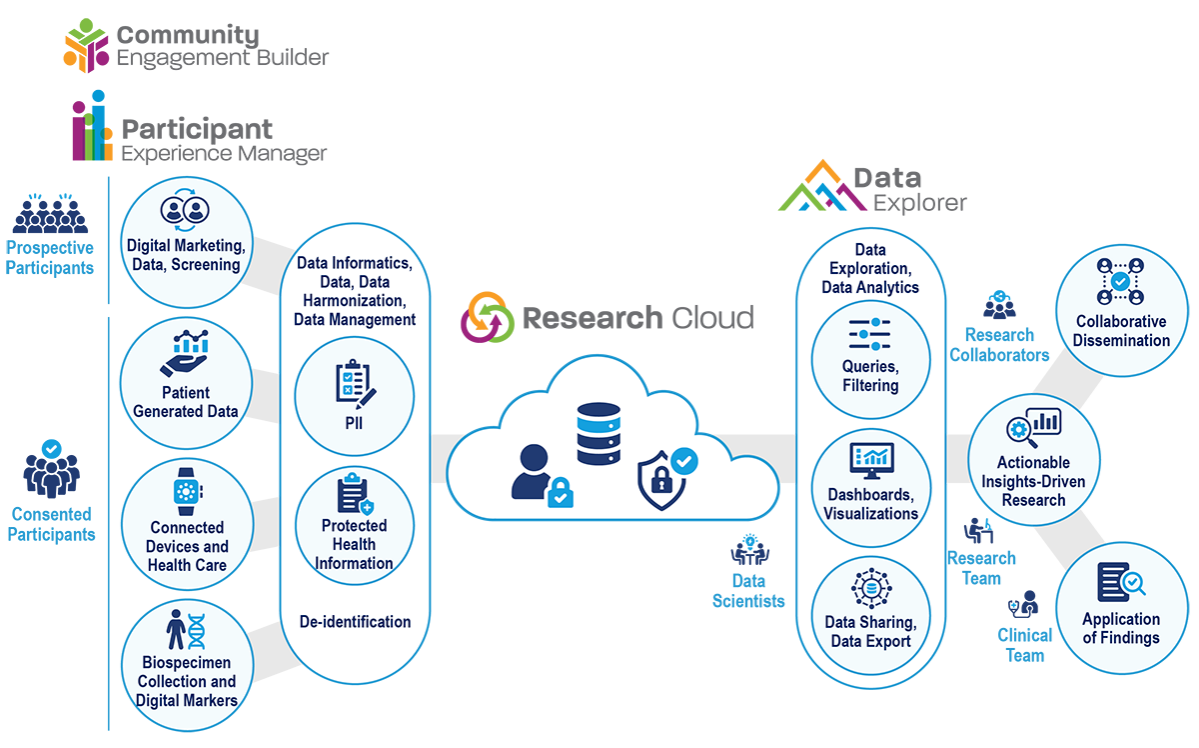
Schematic of the DHRP end-to-end unified data informatics platform and its PXM, RC, and DX tools facilitating collaboration among research teams in a large multisite consortium. DHRP: digital health research platform; DX: Data Explorer; PHI: protected health information; PXM: Participant Experience Manager; RC: Research Cloud.

The technical architecture of the DHRP is highly configurable, using a low-code approach and promoting an open ecosystem without the need for custom software development. The AOU used a range of DHRP tools to facilitate the study: (1) Participant Experience Manager (PXM), (2) Research Cloud (RC), 3) Data Explorer (DX), and (4) Community Engagement Builder (CEB). The DHRP tools are shown in Figure 4.

The DHRP adopted a hybrid technical strategy, blending readily accessible commercial tools and subsystems with customized application layer enhancements and integrations. This approach provided a tailored solution of high-quality explicitly crafted tools that are also generalizable to meet the needs of various study designs. The platform supported an open ecosystem to integrate with additional devices and to embed experimental digital health technology modules within study protocol pathways.

The DHRP used multilayered enterprise architecture to achieve secure storage of data, along with flexibility and scalability. The Cloud Services and Infrastructure Management Layer provided dynamic autoscaling to meet the changing demands of studies over time. The Data Lake and Database Layer managed and stored large volumes of multisource data, including participant data and operational data. Key functions of the DHRP included data integration, data transformation, advanced analytics, data governance, real-time data processing, and metadata management. The platform used centralized cloud-hosted databases (MySQL, Snowflake [NoSQL], Redshift) for secure data storage, and a data warehouse. The Functional Modules Layer facilitated interconnection of the DHRP to multiple third-party application programming interfaces (APIs) and end-user applications.

The DHRP used several methodological testing frameworks and capabilities, as well as scientific testing support protocols, including user research, functional randomization testing, randomization for scientific testing, survey response randomization, and bias mitigation. In addition, Agile software development life cycle (SDLC) and continuous integration/continuous delivery (CI/CD) processes facilitated the delivery of high-quality enterprise software. The “shift-left” SDLC reduced the complexity of software development, thus enabling the study to respond quickly to protocol amendments without disrupting the participant experience. Examples of configuration updates include the addition of new surveys, workflow changes, or revision of study-related content.

Containerization and microservices methods were applied in the DHRP to optimize flexibility, scalability, efficiency, system performance, security, and privacy. Containerization provides a standardized, efficient approach to software deployment by encapsulating applications within isolated environments. This ensures consistent performance and compatibility across diverse computing platforms, which is essential for reproducible and scalable scientific research [24]. Microservices architecture breaks down complex software into smaller, independent parts that work together, making it easier to update and scale applications. DHRP microservices are described in Table 1.

**Table 1:** Microservices used in the DHRP^a^.

Microservice	Function
Ancillary study integration	Facilitates integration with third-party study tools
Appointment management	Schedule, view, and update appointments with maps and geolocation
Asynchronous messaging	Advanced data synchronization service enabling large-scale cross-platform integration
Authentication services	Enables authentication and single sign-on
Case management	Create cases with specific actions to take for a group of participants
Communications services	Transmit targeted email or SMS text or push communications to mobile apps
Comparative insights	Provide demographic-based results (by age, location, etc) for survey responses
Dashboards	Canned visualizations of protocol activities to assist research staff in overseeing program activities; use default dashboards or create your own customized dashboard
Data integration	Integrate with an external data hub
Data management	Scalable storage of large volumes of multisource data
Data retrieval services, multiple	Retrieve data from various sources: Medicare, Epic, Cerner, Athena Health, Fitbit, Apple HealthKit
Deep linking	Communications (email and SMS text) linked to specific steps in the participant workflow to improve participant engagement
eConsents^b^ and agreements	Manages primary and additional consent agreement modules
File-sharing services	Ability to manage upload and sharing of test results with participants
Fulfillment services	Order management and delivery tracking of biosample kits and other assets required to support data collection
Identity verification	Secure, remote verification of participants for account access
Insights	Search, analyze, and visualize data about participants and study activities; ability to create custom dashboards, reports, and data exports based on the unique needs of a study
Mobile apps	Study branded mobile apps for participant access; supports iOS and Android clients and adaptive web for participants choosing not to use mobile apps
No-login experiences	Enable participants to complete survey activities without authentication
Reports and exports	Create customized reports and exports for use with study engagement activities
Segmentation	Create complex filtering criteria to identify a group of participants to target for a protocol activity and engagement and may be used to create a case or transmit communications
Survey import	Import survey definitions and participant survey data from third party–hosted survey platforms
Task management	Create and view appointments, cases, and follow ups
Time-based event management	Create user engagement workflows based on time-based protocol requirements
UX^c^ and advance workflow customization	Enable the creation of simplified user engagements and support for complicated time- and event-driven workflow; includes configurable page and navigation items, business rules, and conditions

^a^DHRP: digital health research platform.

^b^eConsent: electronic informed consent.

^c^UX: user experience.

#### DHRP Participant Experience Tool

The PXM facilitated all participant-facing study protocol activities, including (1) electronic informed consent (eConsent), (2) study data collection and data sharing, and (3) secure bidirectional communication. The PXM operates in English and Spanish and can be translated to more than 30 additional languages. Table 2 describes the third-party services that were integrated with the DHRP to support PXM functionality.

The eConsent functionality, content, and UX were developed to accommodate seventh-grade or lower literacy levels through collaborative UX research with participants, researchers, and NIH staff. The key functionality of eConsent included (1) web-based and mobile device accessibility, (2) built-in knowledge reinforcement, and (3) content accessible by text, audio, or video.

The PXM allowed participants to securely access individual participant portals to complete study-related data collection activities, including submission of survey responses, scheduling appointments to complete study measures, and communication with research staff, as shown in Figure 4. The PXM supported both “repeated surveys” and “longitudinal data collection” with defined events, including complex window management that made certain study tasks available in the participant dashboard during the appropriate window of time. Participants were able to securely review their personal data and genetic results through the PXM.

**Figure 5:**
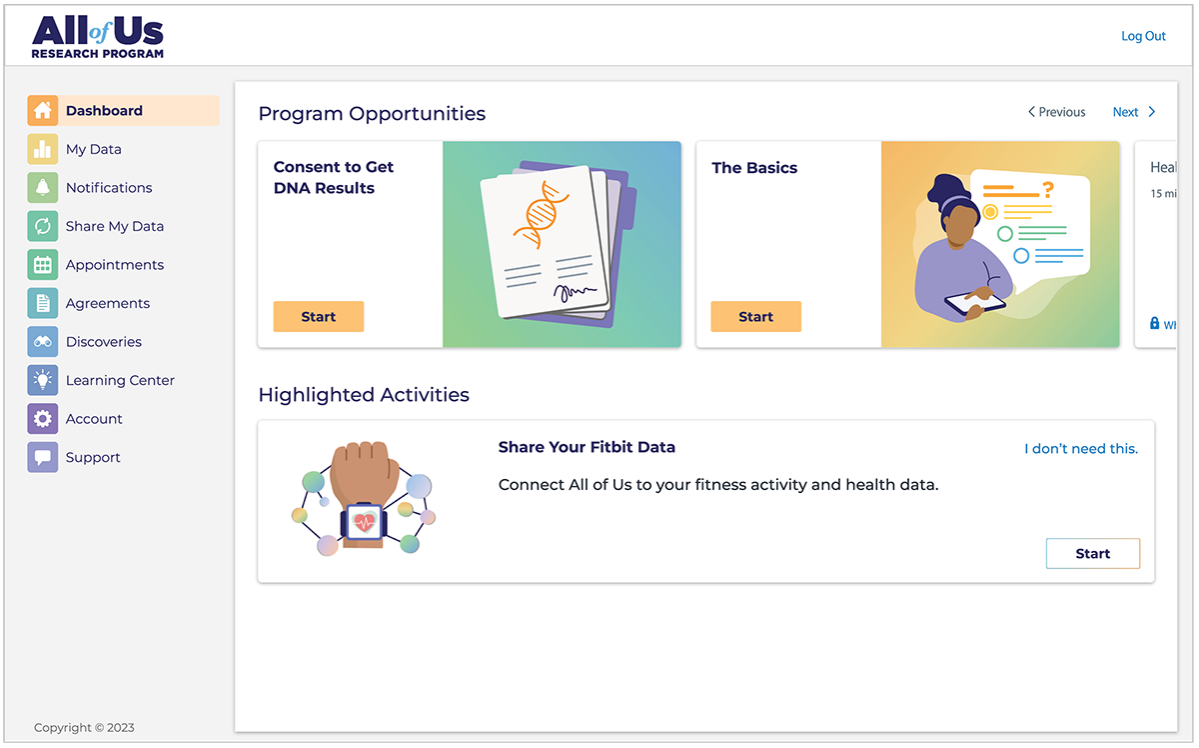
Screenshot of the PXM dashboard. PXM: Participant Experience Management.

The PXM was integrated with a range of third-party fast health care interoperability resource (FHIR) systems enabling participants to share EHRs and medical claims data to contribute to the study. Over the course of the AOU, multiple approaches were also used to facilitate the collection of wearable data from participants—all of which made use of the PXM. These included (1) providing opt-in for self- managed (ie, “bring your own device”) data contributions using participant-owned Fitbit wearables, Google Fit, and Apple HealthKit; (2) providing Fitbit devices directly to participants to encourage the contribution of data; and (3) providing supportive messaging to participants who were provided with Fitbits to both encourage and monitor the optimal approaches to acquiring wearable and related digital health data. This secure, participant-focused design fostered ease-of-use throughout the study (Figure 5).

As part of an end-to-end readiness assessment for pediatric enrollment, the PXM supports engaging with parents of eligible children as proxy users. Parents of children under the age of 5 years old were invited to enroll their children in the study and were authorized to complete permission, authorization, and data collection modules on behalf of their children [25]. Tools enable study staff to engage with children for biosample and physical measurement collection processes using the PXM. Parents can enroll multiple children and select those children in a drop-down menu. As the AOU expands its pediatric recruitment processes, the PXM will support additional parental roles and permissions, as needed, for participants between the ages of 5 and 18 years at the time of consent.

**Table 2:** Participant-centric functionality requires scalable integrations with COTS^a^.

Participant-facing tools	COTS
Communication (email, SMS, postal/printed mail)	Iterable, Lob, Twilio
Genomics counseling and return of information	Color
Remote biosample collection for assays and genomic sequencing (saliva, blood, nasal swabs)	DNA Genotek, Mirimus, Molecular Testing Labs (MTL), Tasso, Quest Diagnostics
EHRs^b^	Epic, Cerner, Athena Health, others
Wearables and biometric devices	Fitbit, Apple HealthKit, Apple Watch, Google Fit
Identity verification	ID.me
Data visualization/exploration, dashboards, analytics	Metabase, Tableau, Google Analytics
Surveys, codebooks	REDCap, Qualtrics
Enable aggregate participant data comparisons at scale	Snowflake
Shipment and logistics	US Postal Service, Aftership, FedEx
Participant data privacy and security	Akamai, HackerOne

^a^COTS: commercial-off-the-shelf services.

^b^EHR: electronic health record.

#### DHRP Research Administration Tools

The RC empowers research staff to complete direct engagement, monitoring, and support to assist participants with completion of study activities. The RC enables research staff to communicate with participants via automated digital marketing, email, and SMS text messaging and to securely collect participant data using built-in computer-assisted telephone interviewing (CATI) capabilities. The RC allowed staff to assist participants with account access, scheduling of study measures visits (in-clinic or remote), and tracking of participant progress via customizable dashboards. The RC was critical in building relationships to keep participants engaged and ensure that study tasks were completed.

For security and privacy, the RC implemented a Roles-Functions-Permissions-based framework system, which granted limited access to select data and workflows based on the research staff’s assigned role within the platform. The 13 distinct user roles available in the RC include program coordinators, data analysts, research assistants, and system administrators. After completing 2-step authentication login, RC users can review a role-based data dashboard or download participant-level research data. To facilitate adoption, a training environment was created, which allows RC users to practice their workflows without compromising real study data. The RC can also support expansion to ancillary studies and can extend user roles to allow access to multiple projects through 1 dashboard.

#### DHRP Data Management Tool

The DX is a self-service analytics tool that enabled study staff to access structured or unstructured data to construct custom reports, dashboards, and insights into collected data and metadata. The DX allows researchers to review and analyze study data from all sources (surveys, collected biometric measures, and third-party shared data) in real time within 1 dashboard. The DX also supports the use of data analytics tools (RStudio, Python, JupyterHub) within the platform on live data. This capability allows researchers to assess data quality and preliminary study results in real time. Modular data pipelines were developed in Python, allowing for scalability and adaptability to many different data sources and data analytics functions.

#### DHRP Content Management Tool

The content management tool, called CEB, delivered study outreach materials and hosted the study recruitment website. It enabled rapid and low-cost creation and deployment of customized recruitment websites, event marketing, and landing pages tailored to a site’s specific catchment area. The CEB includes UX-validated templates to optimize content with appropriate levels of cultural sensitivity and language translation. The CEB was used to generate websites that educate and recruit people to the AOU or ancillary studies and was also used to build the nationwide recruitment website [26].

The CEB was integrated with the RC to provide real-time enrollment metrics to support strategic adjustment of recruitment strategies for study success. Digital marketing analytics generated by the CEB enabled staff to track conversions from both online (recruitment website, lead generation, events landing pages, and social networking links) and offline (QR codes from posters and flyers) sources. By leveraging these analytics, staff gained insights into the effectiveness of different recruitment methods and made informed decisions to optimize recruitment efforts and meet enrollment and diversity targets.

#### DHRP Research Staff Training and Support Tools

After the study was launched, there was a recognized need to provide comprehensive and efficient support and training to research staff using DHRP tools. The Help Center and the Vibrent Research Academy were digital training resources created to support researchers’ execution of study management and outreach tasks. The Help Center served as a knowledge base for site staff using the RC or CEB tool and provides a detailed explanation of DHRP features, along with supplemental articles, troubleshooting tips, and other resources. The Vibrent Research Academy offers short courses that provide detailed, multimedia demonstrations and instructions on the use of DHRP tools and features.

### Ethical Considerations

#### eConsent Process

Through the PXM, we delivered a modular eConsent process to facilitate a self-paced, dynamic, and adaptive experience for participants [27]. The eConsent process met the regulatory and compliance requirements of all 50 states to support geographic inclusivity and access to participation. A formative consent evaluation module was included to assess participant informedness following a review of each eConsent module. Before providing a digital signature, participants were required to correctly answer 80% of the evaluation questions. Participants were able to withdraw consent from any individual consent module at any time, with the option to reconsent again in the future. Digital copies of completed consent forms were stored in the PXM. This same mechanism also facilitated electronic assent on behalf of eligible minors.

#### Data Security, Encryption, Regulation, and Compliance

All participant-facing materials were approved by a centralized Institutional Review Board. The DHRP maintains a “defense in depth” security posture to keep participant data safe and private. Data security and privacy are implemented in accordance with established industry-standard policies, procedures, and technology system guidelines, such as Federal Information Security Modernization Act (FISMA) and National Institute of Standards and Technology (NIST) Cybersecurity Framework for Security and Privacy Controls (NIST SP 800-53 r5, NIST SP 800-39, NIST SP 800-37) and are Health Insurance Portability and Accountability Act (HIPAA) compliant. Technical safeguards include strong data encryption (AES256, TLS 1.2 and higher) in flight and at rest. The DHRP is compliant with 21 CFR part 11 and ensures entries come from an authenticated and authorized source. Role-based permissions ensure that only authorized users are permitted to change settings.

### Community Engagement

The AOU is a population-based precision medicine initiative operating on a community engagement structure [15,21]. To operationalize the program’s core values, a community-based participatory research approach was implemented to develop a national network of actively engaged community organizations and individuals to build community advisory boards (CABs). CABs collaborated with health care provider organizations (HPOs), local community centers, academic and industry partners to provide study guidance.

Community engagement partners facilitated direct engagements between prospective participants and members of their specific communities, which could take the form of sending structured electronic communications, setting up in-person opportunities at events and physical facilities, promoting by word of mouth, and providing biosample collection opportunities when supported by other program partners. These partners encourage initial enrollment as their primary function.

### Participant Recruitment and Enrollment

Participant recruitment, eligibility, and enrollment were delivered through in-person and digital methods facilitated by the DHRP [15,21]. Outreach was conducted at local community events and conferences, by word of mouth, digital advertising, and physical distribution of study-branded flyers and promotional items. The CEB tool of DHRP provided customized, culturally appropriate recruitment webpages in multiple languages. Participants who enrolled remotely were able to communicate with staff by phone or email via the PXM. Remote participants were also able to use the PXM to locate the nearest site at which to complete their physical study measurements. Fully remote participants could independently complete all activities, including consent, surveys, EHR data sharing, biosample collection, and communication with study staff, without reporting in person to an HPO site.

Participants were also recruited in person at HPO and mobile clinic sites throughout the United States, with enrollment completed in the DHRP, with or without assistance from site staff. A myriad of program partners used the DHRP to support recruitment, enrollment, and retention. Health care–providing organizations, such as large health systems and federally qualified health centers, facilitated both initial recruitment in the same manner as community engagement partners and directly facilitating (1) consent, (2) completion of electronic surveys, (3) completion of biosample collection in both remote and in-person engagements, and (4) EHR data collection, as well as providing human support for all program activities when a program participant was recruited from their patient populations.

### Study Data Collection

All study data were collected in the DHRP. Participants used the PXM tool to submit surveys and complete data sharing from third-party services. To minimize the participant burden, all surveys were available in separate, self-paced modules, allowing participants to save their progress and return to the modules for completion later. To support data collection, research staff used the DX tool to identify participants with incomplete study tasks, such as eConsents or surveys. The RC was then used to develop targeted engagement campaigns to remind participants of any remaining incomplete study tasks requiring attention. A description of the sources and types of data collected in the DHRP is available in Table 3.

**Table 3:** Multisource data types collected in the DHRP^a^.

Data source	Data formats
eConsent^b^ signature	PDF
EHRs^c^	JavaScript Object Notation (JSON)–formatted FHIR^d^ payloads
Consumer wearables (Fitbit, Apple HealthKit)	Apple HealthKit health data: JSON Apple HealthKit EHR data: JSON
Biometric measures	JSON-formatted FHIR payloads
Genetic results	PDF reports
Surveys	JSON-formatted FHIR payloads: questionnaire response
Personally identifiable information	JSON-formatted FHIR payloads
Participant communication	Comma-separated value (CSV), JSON
Participant communication	CSV
Prospective participants	CSV
Cognitive assessments	JSON-formatted FHIR payloads
Pediatric assent and surveys	PDF and JSON-formatted FHIR payloads: questionnaire response
Digital marketing data	CSV, Urchin Tracking Module (UTM) parameters

^a^DHRP: digital health research platform.

^b^eConsent: electronic informed consent.

^c^EHR: electronic health record.

^d^FHIR: fast health care interoperability resource.

## Results

### Participant Characteristics

**Figure 6:**
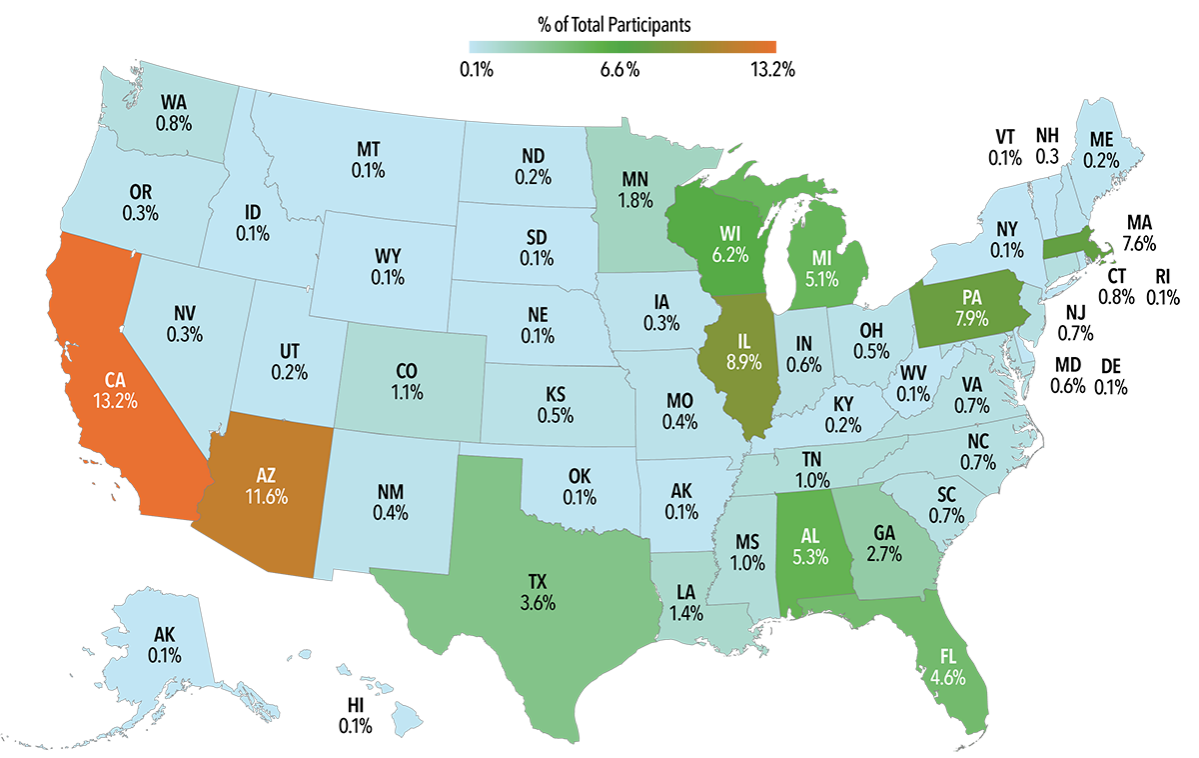
Geographic distribution of AOU participants enrolled through the DHRP by US state as of March 21, 2024. State enrollment varied from 13.2% (n=86,803) in California to 0.1% (n=706) in New York. The western region had the highest enrollment (28.1%). AOU: All of Us Research Program; DHRP: digital health research platform.

The DHRP was launched for use in May 2018. As of April 2024, a total of 705,719 participants had enrolled in the AOU through the DHRP. Of these participants, 74% (n=524,264) completed all study activities in the core study protocol, including all required survey modules. In this cohort, 55% (n=386,377) identified as women, 51% (n=360,303) were aged 35-64 years, and 48% (n=340,423) identified as White. The platform was used by participants and research teams in each of the 50 US states and territories, with 8% (n=58,211) participants representing rural areas (Figure 6).

The AOU used the DHRP to build a diverse research cohort in which 87% (n=457,514) identified as members of groups underrepresented in biomedical research (UBR) in at least 1 demographic category (age, race, sex, gender, income, education, or disability). More specifically, 46% (n=243,296) participants identified as racial minorities, 36% (n=189,087) reported a low income, and 20% (n=104,916) reported a disability. One third of the cohort (n=219,355, 31%) were aged 65 years and over, 8% (n=58,211) identified as rural residents, and 28% (n=197,601) were people with limited access to health care. More than 5% (n=~37,000) of all program participants opted to participate in the program in Spanish. UBR diversity metrics of the cohort are available in Table 4.

**Table 4:** AOU^a^ participants’ (N=705,719) sociodemographic characteristics as of April 2024.

Characteristics		Participants, n (%)
**Age group (years)**		
	18-24	25,707 (3.6)
	25-44	213,958 (30.3)
	45-64	246,699 (35.0)
	65-84	205,072 (29.0)
	≥85	14,283 (2.0)
**Gender**		
	Man	237,070 (33.6)
	Woman	386,377 (54.7)
	Nonbinary	2729 (0.4)
	Transgender	1169 (0.2)
	Other/multiple selections	4010 (0.5)
	Unspecified/no answer	74,364 (10.5)
**Racial identity**		
	White	340,423 (48.2)
	Black or African American	102,371 (14.5)
	Asian	22,281 (3.2)
	Multirace/other	55,872 (7.9)
	None/no answer	86,477 (12.3)
**Education**		
	Less than high school	51,236 (7.3)
	Grade 12 or General Educational Development (GED)	113,545 (16.1)
	College, 1-3 years	165,317 (23.4)
	College, 4 years or more	148,910 (21.1)
	Advanced degree	42,504 (20.2)
	Unspecified/no answer	84,207 (11.9)
**Annual household income (US $)**		
	≤24,999	143,939 (20.1)
	25,000-49,999	96,800 (16.0)
	50,000-74,999	70,599 (10.0)
	75,000-99,999	55,0752 (7.8)
	100,000-199,000	101,965 (14.4)
	≥200,000	45,136 (6.4)
	Unspecified/no answer	188,205 (26.7)
**Rurality**		
	Rural	58,211 (8.2)
	Nonrural (metro/suburban)	644,852 (91.4)
	Unspecified/no answer	2656 (0.4)

^a^AOU: All of Us Research Program.

### Data and Tool Usage

Approximately 4,500,000 combined survey and eConsent modules have been completed through the DHRP. Analysis of eConsent formative evaluation questions revealed that after reviewing eConsent modules, over 95% (n=670,433) of the participants were able to differentiate the research program from medical care, understand the voluntary nature of their involvement, and comprehend their right to withdraw at any point in time [28]. Complete data collection metrics are in Table 5.

**Table 5:** Data collected and tool usage metrics from the DHRP^a^ for the AOU^b^.

Measures		Data, n
**Participant data sources**		
	Fitbit	49,627
	Apple HealthKit	19,940
	EHRs^c^	14,431
	Total survey modules completed	3,685,608
	Total physical biometric study measures	538,160
	Saliva kits	59,819
**eConsent^d^**		
	Total primary eConsent modules completed	2,015,622
	EHR eConsent module	632,644
	Genomics return of results eConsent module	406,781
	Cognitive assessment eConsent module	85,572
**Digital marketing**		
	Prospective participants contacted	378,010
	Automated email and SMS text campaigns delivered	3677
	Custom HTML templates created by sites in the partner CEB^e^ tool	1838
	Multichannel communication automations (bilingual)	≥300
**Participant multimodal communications**		
	Emails sent to participants	64,837,083
	SMS text messages sent to participants	7,794,054
	Mobile app push notification messages sent to participants	463,975
	Direct mail sent to participants	1,531,814
	Site staff remote engagement with participants	1,168,079
	Protocol step completions logged by site staff due to remote engagements	412,026
	CATI^f^ sessions	42,795
	Appointments scheduled (remote and site based)	301,393

^a^DHRP: digital health research platform.

^b^AOU: All of Us Research Program.

^c^EHR: electronic health record.

^d^eConsent: electronic informed consent.

^e^CEB: Community Engagement Builder.

^f^CATI: computer-assisted telephone interviewing.

**Figure 7:**
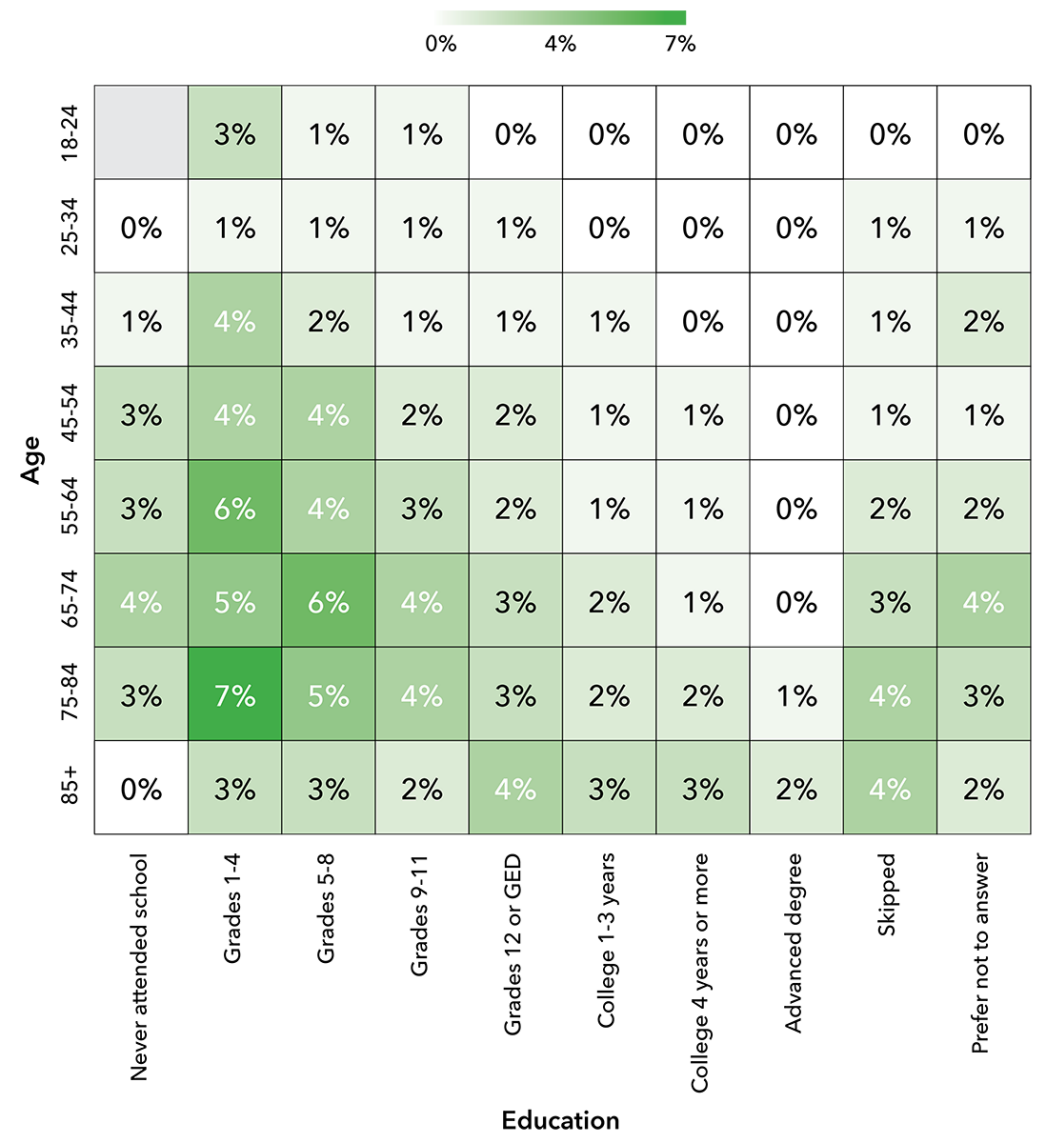
Heat map showing cross-sectional use of CATI by age and education level. CATI: computer-assisted telephone interviewing; GED: General Educational Development.

**Figure 8:**
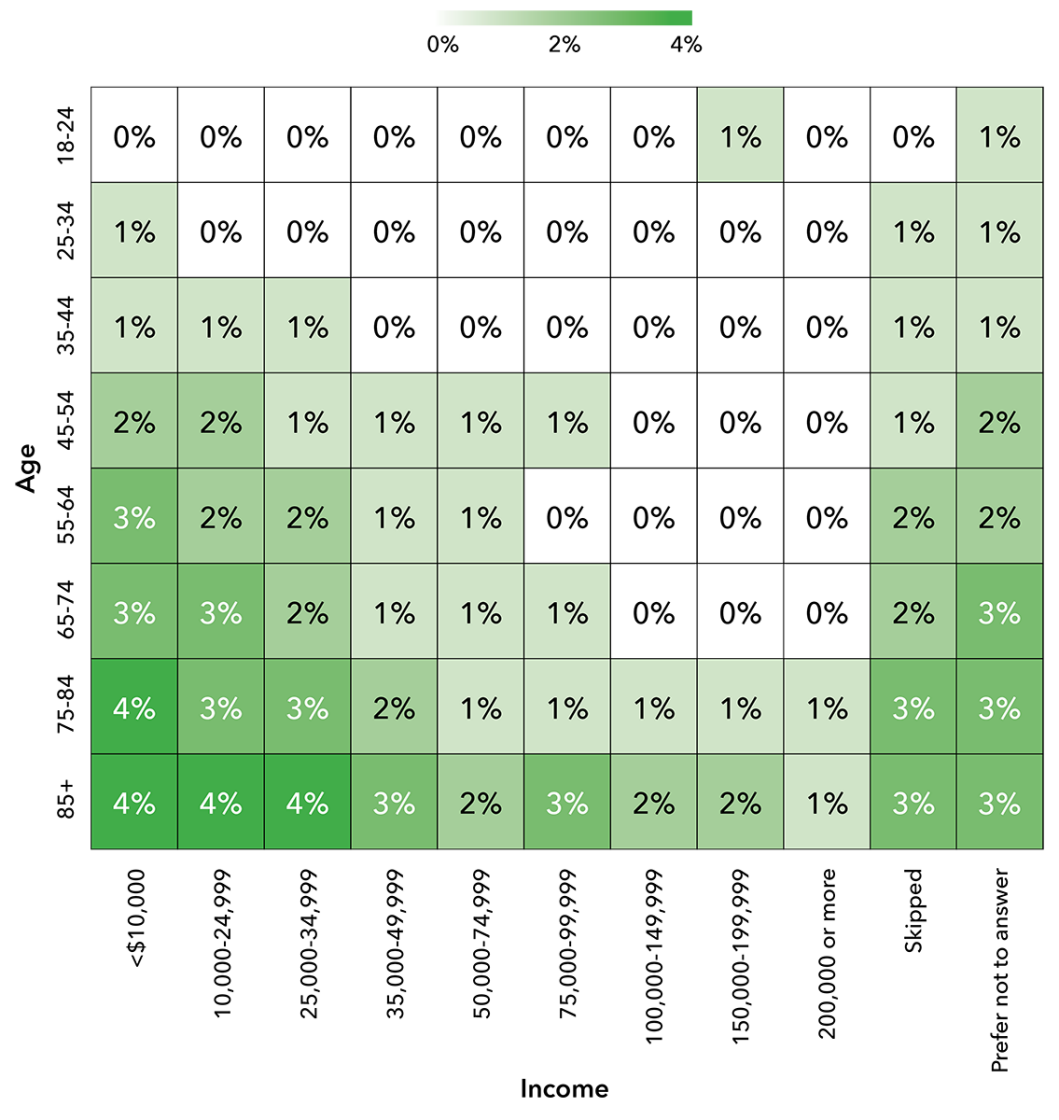
Heat maps showing cross-sectional use of CATI by age and income. CATI: computer-assisted telephone interviewing.

**Figure 9:**
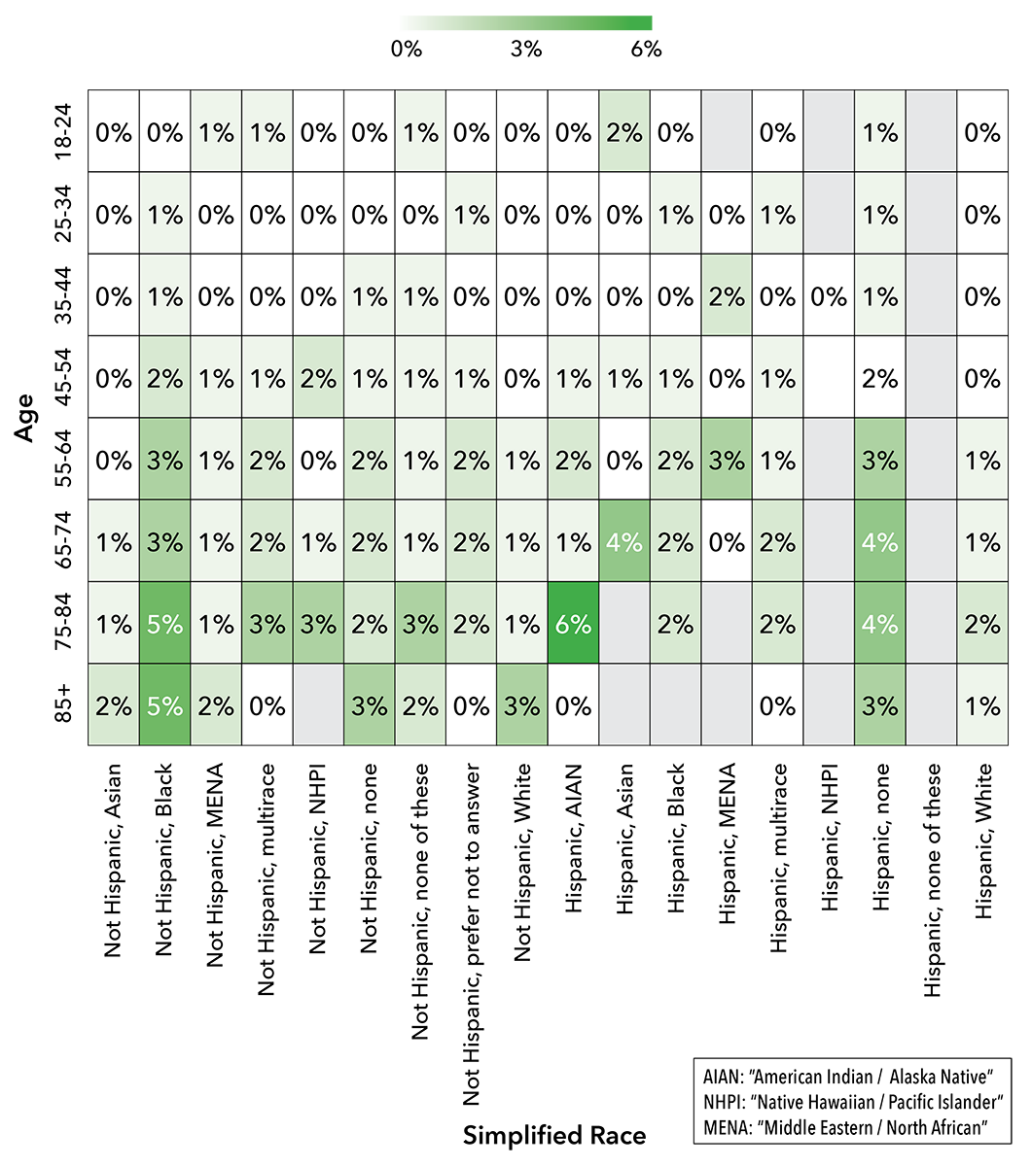
Heat maps showing cross-sectional use of CATI by age, race, and ethnicity. AIAN: American Indian or Alaska Native; CATI: computer-assisted telephone interviewing; NHPI: Native Hawaiian or Pacific Islander; MENA: Middle Eastern or North African.

The use of CATI capabilities within the DHRP demonstrated the positive effects of combining digital and in-person engagement methods to enhance the participation of UBR groups in research. Among participants who completed the Social Determinants of Health (SDOH) survey module (n=276,706, 42%, eligible participants) for the study, CATI technology supported significant portions (as high as 47%) of survey completions within cross-sectional UBR groups. Cross-sectional analysis by participant age, education, income, and race-ethnicity showed increased use of CATI-assisted sessions within certain UBR groups. Specifically, the increased use of CATI was evident among those with education levels below grade 9, those with an income below US $35,000/year, people aged 55 years and older, and people identifying as non-Hispanic Black or Hispanic of any race. The cross-sectional use of CATI is shown in Figures 7, 8, and 9. Those with lower education levels, of older age, with lower incomes, or from racial and ethnic minorities are recognized as UBR populations [29].

Another demonstration of the effective engagement capabilities of the DHRP was found in the completion rates of the optional COVID-19 Pandemic Evaluation (COPE) survey. The COPE survey was added in response to the COVID-19 pandemic, which was first confirmed in the United States in January 2020. The COPE survey was launched in the DHRP in May 2020, and 6 iterations were developed, modified, and released to participants through February 2021. A previous study of the COPE survey showed that the modification of surveys led to a significant increase (*P*<.001) in the participant response rate over 10 months of survey delivery [30]. Overall, completion rates for the COPE survey among individuals enrolled under UBR categories showed an increase from 11% in the first survey to 16% in the final survey. These findings underscore the significance of using a digital platform able to accommodate rapid implementation of protocol amendments.

## Discussion

### Principal Findings

The design and implementation of the DHRP in this study demonstrates that a DHRP with technical architecture for participant-focused design can effectively be used to recruit, engage, and collect longitudinal health data from participants with geographic, racial, and sociodemographic diversity. The collaborative, community-informed approach used to develop the DHRP, combined with validation through UX research, contributed to its effective design for use in diverse populations [31]. The participant-centric design of the DHRP may be broadly applicable to increase community engagement of people from diverse backgrounds in other research study designs, including cross-sectional, case-control, and experimental clinical trials [32].

The principles of generalizability, usability, cultural sensitivity, and accessibility were consistently implemented throughout the human-centered design and building of the DHRP for this study [33]. The diversity of participant users, combined with the high eConsent completion rates, support the idea that the PXM is, in fact, participant friendly, accessible, and usable for various groups, as intended. The use of the DHRP’s CATI technology in this study also demonstrates the successful use of digitally enabled, human-delivered engagement methods to enhance participation of UBR groups. In this study, CATI enhanced data collection and increased study completion rates of the SDOH survey module among people with lower education levels, those with lower incomes, older people, and those from racial and ethnic minority groups. This shows that the DHRP can be used to increase cohort diversity and build trust with UBR groups [34]. In addition, the increase in engagement demonstrated by the COPE survey results shows that the researcher tools, RC and CEB, are integral to facilitating participant engagement within DHRP. Furthermore, integrating the participant-facing tool (PXM) with the researcher tools (RC and CEB) in a containerized manner protected participant autonomy and privacy, while also maintaining workflow automation and bidirectional communication between participants and study staff.

From an engagement perspective, it was important to establish a standardized eConsent process that was accessible and comprehensive for all individual platform users regardless of their geographical location, method of enrollment, preferred language, educational level, digital literacy, or digital device used [27]. PXM eConsent modules were successfully used with high participant informedness demonstrated by high rates of completion of the formative consent evaluation. Well-designed and well-delivered eConsent provides transparency to participants at study initiation and can increase volunteerism, participant understanding of research, and participant satisfaction [28]. This can lead to increased trust and future participation in research among groups historically underrepresented in the field [27,35]. This result supports the idea that this digital research platform can enhance the delivery of longitudinal studies and can be used by participants with different levels of digital literacy.

### Strengths and Limitations

This novel study has several strengths. This demonstration helps shed light on how digital research platforms, such as the DHRP, can be effectively used to increase the participation of UBR groups. The collaborative approach used in the design of the DHRP ensured the platform was able to meet the needs of people from various sociodemographic backgrounds and with varying levels of comfort with digital technology. The comprehensive design of the DHRP tools appears to have decreased participant barriers to research participation commonly reported in the literature [36]. These aspects should be further investigated in future research.

Although this study has numerous strengths, it also presents potential limitations. Although the cohort is 8% rural (n=58,211), it does not fully represent the current US population, which is 13.8% rural by county definition [37]. This limitation is a function of the overall AOU research processes that determine the geographic distribution of participants based on the availability of local affiliated recruitment sites and is not a direct limitation of the DHRP itself.

Another possible limitation of this study is the lack of available data with which to evaluate the use of the DHRP within the pediatric population. This is a result of the AOU being in its initial phases of enrolling pediatric participants. Although the DHRP was designed and built to accommodate the complexities of consenting, engaging, and collecting data for pediatric research, we currently do not have adequate data to conduct a conclusive analysis of the efficacy of the DHRP within this group. Therefore, we are unable to report on potentially unforeseen challenges related to the use of the DHRP in pediatric research, as they have not yet been encountered in this study.

### Conclusion

We created and validated a digital research platform to support the development of a large, nationwide, community-engaged, longitudinal cohort study (AOU) of diverse participants from UBR groups. This study demonstrates the promising potential of digital platforms to enable the curation, harmonization, and management of high-volume longitudinal multisource data sets from diverse populations for precision medicine research. Digital platforms may provide solutions to researchers who aim to broaden their catchment area in site-based, decentralized, and hybrid research methods and support the development of more diverse research cohorts. With continued development, digital research platforms, such as the DHRP described here, may lead to the successful establishment of best approaches to engage health-disparate, minority, vulnerable, and other UBR populations into clinical research to broadly improve the overall health of all communities.

## Abbreviations

AOU: All of Us Research Program
CAB: community advisory board
CATI: computer-assisted telephone interviewing
CEB: Community Engagement Builder
COPE: COVID-19 Pandemic Evaluation
COTS: commercial-off-the-shelf services
DHRP: digital health research platform
DX: Data Explorer
eConsent: electronic informed consent
EHR: electronic health record
FHIR: fast health care interoperability resource
HPO: health care provider organization
NIH: National Institutes of Health
NIST: National Institute of Standards and Technology
PXM: Participant Experience Manager
RC: Research Cloud
SDLC: software development life cycle
SDOH: Social Determinants of Health
UBR: underrepresented in biomedical research
UX: user experience

## References

[1] Caruana EJ, Roman M, Hernández-Sánchez J, Solli P (2015). Longitudinal studies. J Thorac Dis.

[2] Popejoy AB, Fullerton SM (2016). Genomics is failing on diversity. Nature.

[3] Allison K, Patel D, Kaur R (2022). Assessing multiple factors affecting minority participation in clinical trials: development of the Clinical Trials Participation Barriers Survey. Cureus.

[4] Hamel LM, Penner LA, Albrecht TL, Heath E, Gwede CK, Eggly S (2016). Barriers to clinical trial enrollment in racial and ethnic minority patients with cancer. Cancer Control.

[5] Ejiogu N, Norbeck JH, Mason MA, Cromwell BC, Zonderman AB, Evans MK (2011). Recruitment and retention strategies for minority or poor clinical research participants: lessons from the Healthy Aging in Neighborhoods of Diversity across the Life Span study. Gerontologist.

[6] Allmark P (2004). Should research samples reflect the diversity of the population?. J Med Ethics.

[7] Gross AS, Harry AC, Clifton CS, Della Pasqua O (2022). Clinical trial diversity: an opportunity for improved insight into the determinants of variability in drug response. Br J Clin Pharmacol.

[8] Clark LT, Watkins L, Piña IL, Elmer M, Akinboboye O, Gorham M, Jamerson B, McCullough C, Pierre C, Polis AB, Puckrein G, Regnante JM (2019). Increasing diversity in clinical trials: overcoming critical barriers. Curr Probl Cardiol.

[9] McConnell MV, Shcherbina A, Pavlovic A, Homburger JR, Goldfeder RL, Waggot D, Cho MK, Rosenberger ME, Haskell WL, Myers J, Champagne MA, Mignot E, Landray M, Tarassenko L, Harrington RA, Yeung AC, Ashley EA (2017). Feasibility of obtaining measures of lifestyle from a smartphone app: the MyHeart Counts Cardiovascular Health Study. JAMA Cardiol.

[10] Spartano NL, Lin H, Sun F, Lunetta KL, Trinquart L, Valentino M, Manders ES, Pletcher MJ, Marcus GM, McManus DD, Benjamin EJ, Fox CS, Olgin JE, Murabito JM (2019). Comparison of on-site versus remote mobile device support in the Framingham Heart Study using the Health eHeart Study for Digital Follow-up: randomized pilot study set within an observational study design. JMIR Mhealth Uhealth.

[11] Hwang DA, Lee A, Song JM, Han H (2021). Recruitment and retention strategies among racial and ethnic minorities in web-based intervention trials: retrospective qualitative analysis. J Med Internet Res.

[12] Dockendorf MF, Hansen BJ, Bateman KP, Moyer M, Shah JK, Shipley LA (2021). Digitally enabled, patient-centric clinical trials: shifting the drug development paradigm. Clin Transl Sci.

[13] Greenhalgh T, Wherton J, Papoutsi C, Lynch J, Hughes G, A'Court C, Hinder S, Fahy N, Procter R, Shaw S (2017). Beyond adoption: a new framework for theorizing and evaluating nonadoption, abandonment, and challenges to the scale-up, spread, and sustainability of health and care technologies. J Med Internet Res.

[14] Nebeker C, Murray K, Holub C, Haughton J, Arredondo EM (2017). Acceptance of mobile health in communities underrepresented in biomedical research: barriers and ethical considerations for scientists. JMIR Mhealth Uhealth.

[15] Denny JC, Rutter JL, Goldstein DB, Philippakis A, Smoller JW, Jenkins G, Dishman E, All of Us Research Program Investigators (2019). The "All of Us" Research Program. N Engl J Med.

[16] Armenta A, Serrano A, Cabrera M, Conte R (2011). The new digital divide: the confluence of broadband penetration, sustainable development, technology adoption and community participation. Inf Technol Dev.

[17] Anderson-Lewis C, Darville G, Mercado RE, Howell S, Di Maggio S (2018). mHealth technology use and implications in historically underserved and minority populations in the United States: systematic literature review. JMIR Mhealth Uhealth.

[18] Mapes BM, Foster CS, Kusnoor SV, Epelbaum MI, AuYoung M, Jenkins G, Lopez-Class M, Richardson-Heron D, Elmi A, Surkan K, Cronin RM, Wilkins CH, Pérez-Stable EJ, Dishman E, Denny JC, Rutter JL, All of Us Research Program (2020). Diversity and inclusion for the All of Us research program: a scoping review. PLoS One.

[19] Ghadessi M, Di J, Wang C, Toyoizumi K, Shao N, Mei C, Demanuele C, Tang R, McMillan G, Beckman RA (2023). Decentralized clinical trials and rare diseases: a Drug Information Association Innovative Design Scientific Working Group (DIA-IDSWG) perspective. Orphanet J Rare Dis.

[20] Nijhawan LP, Janodia MD, Muddukrishna BS, Bhat KM, Bairy KL, Udupa N, Musmade PB (2013). Informed consent: issues and challenges. J Adv Pharm Technol Res.

[21] All of Us Research Program overview. National Institutes of Health.

[22] Doerr M, Grayson S, Moore S, Suver C, Wilbankson J, Wagner J (2019). Implementing a universal informed consent process for the All of Us Research Program. Biocomputing.

[23] Participant Technology Systems Center. National Institutes of Health.

[24] Casalicchio E, Iannucci S (2020). The state‐of‐the‐art in container technologies: application, orchestration and security. Concurrency Comput.

[25] All of Us Research Program protocol. National Institutes of Health.

[26] All of Us Research Program. National Institutes of Health.

[27] Doerr M, Moore S, Barone V, Sutherland S, Bot BM, Suver C, Wilbanks J (2021). Assessment of the All of Us research program’s informed consent process. AJOB Empir Bioeth.

[28] Earl CE, Penney PJ (2001). The significance of trust in the research consent process with African Americans. West J Nurs Res.

[29] (2019). Notice of NIH's interest in diversity (NOT-OD-20-031). National Institutes of Health.

[30] Schulkey CE, Litwin TR, Ellsworth G, Sansbury H, Ahmedani BK, Choi KW, Cronin RM, Kloth Y, Ashbeck AW, Sutherland S, Mapes BM, Begale M, Bhat G, King P, Marginean K, Wolfe KA, Kouame A, Raquel C, Ratsimbazafy F, Bornemeier Z, Neumeier K, Baskir R, Gebo KA, Denny J, Smoller JW, Garriock HA (2023). Design and implementation of the All of Us Research Program COVID-19 Participant Experience (COPE) Survey. Am J Epidemiol.

[31] Skarlatidou A, Ponti M, Sprinks J, Nold C, Haklay M, Kanjo E (2019). User experience of digital technologies in citizen science. JCOM.

[32] Guarino J, Parvanova I, Finkelstein J (2022). Characteristics of electronic informed consent platforms for consenting patients to research studies: a scoping review. Stud Health Technol Inform.

[33] Chen E, Leos C, Kowitt SD, Moracco KE (2020). Enhancing community-based participatory research through human-centered design strategies. Health Promot Pract.

[34] Peterson R, Hedden SL, Seo I, Palacios VY, Clark EC, Begale M, Sutherland S, Givens B, McQueen M, McClain JJ (2024). Rethinking data collection methods during the pandemic: development and implementation of CATI for the All of Us Research Program. J Public Health Manag Pract.

[35] Tan RKJ, Wu D, Day S, Zhao Y, Larson HJ, Sylvia S, Tang W, Tucker JD (2022). Digital approaches to enhancing community engagement in clinical trials. NPJ Digit Med.

[36] Heffernan ME, Barrera L, Guzman ZR, Golbeck E, Jedraszko AM, Hays PT, Herzog KA, D'Aquila RT, Ison MG, McColley SA (2023). Barriers and facilitators to recruitment of underrepresented research participants: perspectives of clinical research coordinators. J Clin Transl Sci.

[37] Davis JC, Cromartie J, Farrigan T, Genetin B, Sanders A, Winikoff JB (2023). Rural America at a glance. U.S. Department of Agriculture.

